# Difference in characteristics and outcomes between medullary breast carcinoma and invasive ductal carcinoma: a population based study from SEER 18 database

**DOI:** 10.18632/oncotarget.8142

**Published:** 2016-03-17

**Authors:** Xiao-Xiao Wang, Yi-Zhou Jiang, Xi-Yu Liu, Jun-Jing Li, Chuan-Gui Song, Zhi-Ming Shao

**Affiliations:** ^1^ Department of Breast Surgery, Affiliated Union Hospital, Fujian Medical University, Fuzhou, China; ^2^ Department of Breast Surgery, Key Laboratory of Breast Cancer, Fudan University Shanghai Cancer Center, Shanghai Medical College, Fudan University, Shanghai, China

**Keywords:** medullary breast carcinoma, invasive ductal carcinoma, breast cancer-specific survival, overall survival

## Abstract

Medullary breast carcinoma (MBC) is a unique histological subtype of breast cancer. Our study was designed to identify difference in characteristics and outcomes between MBC and invasive ductal carcinoma (IDC), and further confirm the prognostic factors of MBC. Utilizing Surveillance, Epidemiology, and End Results (SEER), we identified 84,764 eligible patients, including 309 MBC and 84,455 IDC. Compared with the IDC group, the MBC group was associated with younger age at diagnosis, higher grade, more advanced stage, larger tumor size, and higher proportion of triple-negative breast cancer (TNBC). Kaplan-Meier analysis and univariate Cox proportional hazard regression model showed that patients with IDC had significantly better breast cancer-specific survival (BCSS) compared to MBC, but they had similar overall survival (OS). However, MBC histology was no longer a surrogate for worse BCSS or OS after 1:1 matching by age, American Joint Committee on Cancer (AJCC) stage, grade and breast subtype. In addition, it was exposed that not married status, high grade, large tumor size, positive nodal status, the subtype of TNBC and no receipt of radiation therapy were significantly associated with poor BCSS and OS. In conclusion, MBC demonstrated more aggressive behavior but similar outcomes compared to IDC, which may be determined by prognostic factors such as breast subtype. These results not only confer deeper insight into MBC but contribute to individualized and tailored therapy, and thereby may improve clinical management and outcomes.

## INTRODUCTION

Medullary breast carcinoma (MBC) accounts for less than 5% of all invasive breast cancers. The clinicopathological characteristics and outcomes in MBC make it unique from infiltrating ductal carcinoma-not otherwise specified (IDC-NOS) [1].

MBC is characterized by a young age, a large tumor size, a high nuclear grade [2–4]. And some studies found that the MBCs seemed to exhibit a significantly higher proportion of triple-negative phenotype (absence of estrogen receptor (ER), progesterone receptor (PR), and human epidermal growth factor receptor-2 (HER-2)) based on a gene expression analysis or immunohistochemical staining [5, 6]. As is known to all, triple-negative breast cancer (TNBC) usually is relevant to a worse prognosis. However, though patients with MBC show aggressive histological features, there is no consensus regarding difference of clinicopathological characteristics and outcomes between MBC and IDC. Some studies have revealed that MBC histology is associated with a favorable prognosis [2, 5, 7–9]. Other studies did not confirm this conclusion and even some indicated the prognosis of MBC did not differ from IDC [10–12].

Identification of prognostic factors is important for MBC patients so as to not only dictate diagnosis but also provide more appropriate treatment. Martinez et al. reported that in multivariate analysis, advanced age, black race, regional metastases, distant metastases, increased tumor size, ER positivity and increased lymph node metastasis (LNM) were associated with decreased overall survival in a cohort of MBC patients [13]. Park et al. identified there was no difference in disease-free survival (DFS) or overall survival (OS) among MBC patients according to tumor size, hormone receptor status, HER2 status, or adjuvant treatment but patients with lymph nodes metastasis presented a worse DFS and OS [12]. Based on small numbers of patients, different populations of studies and lacked adjustment of confounds, these prognostic factors specific to MBC are relatively unclear.

Given the controversial data on the prognosis of MBC, this study was designed to identify difference in characteristics and outcomes between MBC and IDC, and further confirm the factors related to prognosis of MBC by utilizing Surveillance, Epidemiology, and End Results (SEER), a large population-based database.

## RESULTS

### Demographics and clinical characteristics of study population

A total of 84,764 patients met the eligibility criteria for our study, including 309 (0.36%) MBC patients and 84,455 (99.64%) IDC patients. Table 1 summarizes all of the demographic and clinical characteristics by histological subtype. There were significant differences in the characteristics including age of diagnosis, race, grade, American Joint Committee on Cancer (AJCC) stage, tumor size and breast subtype. MBC patients showed a younger age at diagnosis (42.1% vs. 24.0%, *P* < 0.001) and a poorer grade (grade III and IV, 93.5% vs. 36.2%, *P* < 0.001) than IDC patients. In addition, MBCs were much more likely to have a significantly higher proportion of black race (23.3% vs. 10.4%, *P* < 0.001) and the AJCC stage of II (52.8% vs. 33.8%, *P* < 0.001) than IDCs. Compared with IDC patients, MBC patients had larger tumor size (more tumors > 2 cm and ≤ 5 cm in size, 50.2% vs. 29.2%, *P* < 0.001). With regard to breast subtype, the MBC patients seemed more inclined to present TNBC compared with the IDC patients (56.0% vs. 13.1%, *P* < 0.001). The other tumor characteristics, including married status, laterality, lymph node (LN) status, types of surgery and radiation therapy were similarly distributed between the two histological types.

**Table 1:** Characteristics of patients with medullary breast carcinoma and invasive ductal carcinoma

Characteristics		MBC (*n* = 309)		IDC (*n* = 84455)		Total (*n* = 84764)		*P*^c^
		No.	%	No.	%	No.	%	
**Median follow-up (months) (IQ**R)		17 (9–27)		16 (8–25)		16 (8–25)		
**Age (years)**	**18–49**	130	42.1	20297	24.0	20427	24.1	< 0.001
	**50–79**	179	57.9	64158	76.0	64337	75.9	
Race	**White**	213	68.9	67456	79.9	67668	79.8	**< 0.001**
	**Black**	72	23.3	8800	10.4	8872	10.5	
	**Other**^a^	24	7.8	8199	9.7	8223	9.7	
**Marital statu**s	**Married**	185	59.9	52200	61.8	52385	61.8	0.484
	**Not married**^b^	124	40.1	32255	38.2	32379	38.2	
**Laterali**ty	**Left**	150	48.5	42741	50.6	42891	50.6	0.469
	**Right**	159	51.5	41714	49.4	41873	49.4	
Grade	**I**	2	0.6	18452	21.8	18454	21.8	**< 0.001**
	**II**	18	5.8	35405	41.9	35423	41.8	
	**III and IV**	289	93.5	30598	36.2	30887	36.4	
AJCC stage	**I**	126	40.8	48567	57.5	48693	57.4	**< 0.001**
	**II**	163	52.8	28542	33.8	28705	33.9	
	**III**	20	6.5	7346	8.7	7366	8.7	
Tumor size (cm)	**≤ 2**	141	45.6	56254	66.6	56395	66.5	**< 0.001**
	**> 2 and ≤ 5**	155	50.2	24645	29.2	24800	29.3	
	**> 5**	13	4.2	3556	4.2	3569	4.2	
Nodal status	**0**	240	77.7	60090	71.2	60330	71.2	0.087
	**1 to 3**	54	17.5	18459	21.9	18513	21.3	
	**4 to 10**	10	3.2	3998	4.7	4008	4.7	
	**> 10**	5	1.6	1908	2.3	1913	2.3	
**Breast subtype**	**HR+/Her2−**	99	32.0	60267	71.4	60366	71.2	**< 0.001**
	**HR+/Her2+**	10	3.2	9104	10.8	9114	10.8	
	**HR−/Her2+**	27	8.7	4028	4.8	4055	4.8	
	**Triple negative**	173	56.0	11056	13.1	11229	13.2	
**Type of surgery**	**BCS**	174	56.3	48311	57.2	48485	57.2	0.752
	**Mastectomy**	135	43.7	36144	42.8	36279	42.8	
Radiation	**No**	153	49.5	38055	45.1	38208	45.1	0.116
	**Yes**	156	50.5	46400	54.9	46556	54.9	

Abbreviation: MBC, medullary breast carcinoma; IDC, invasive ductal carcinoma; AJCC, American Joint Committee on Cancer; Her2, human epidermal growth factor receptor 2; HR, hormone receptor; BCS, breast conserving surgery; IQR, interquartile range.

a Other includes American Indian/Alaskan native, and Asian/Pacific Islander.

b Not married includes divorced, separated, single (never married), unmarried or domestic partner and widowed.

c *P* value was calculated among all groups by the Chi-square test, and a bold type indicates significance.

### Comparison of survival between MBCs and IDCs

**Figure 1:**
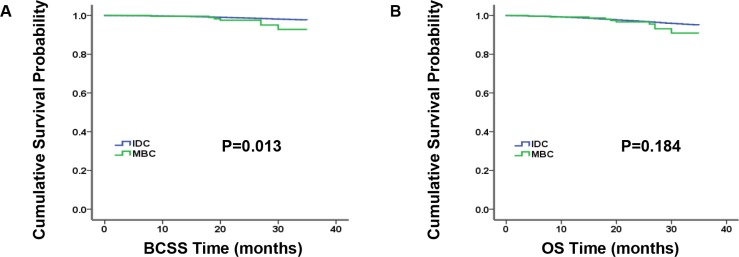
Kaplan-Meier plot and log-rank test compared breast cancer-specific survival (BCSS, A) and overall survival (OS, B) by histology for all patients, medullary breast carcinoma (MBC) vs. invasive ductal carcinoma (IDC)

Kaplan-Meier analysis was used to evaluate breast cancer-specific survival (BCSS) and overall survival (OS) in these two histological types (Figure 1). As it illustrated, patients with IDC had better survival than MBC patients in BCSS (*P* = 0.013), but they had similar OS (*P* = 0.184). In order to further investigate the prognostic factors, the results of the BCSS and OS analyses via univariate and multivariate Cox proportional hazard regression models were demonstrated in Supplementary Table S1 and Table 2, respectively. According to univariate analysis, not married status, grade III and IV, a tumor size of > 5 cm, the increased number of lymph nodes, the subtype of TNBC and no receipt of radiation therapy were significantly associated with poor BCSS and OS, which were validated in the following multivariate analysis. However, after adjusting other prognostic factors, AJCC stage was no longer an independent prognostic factor in multivariate analysis. In the meantime, MBC patients showed significantly worse BCSS than IDC patients (univariate: hazard ratios, HRs = 2.483, 95% confidence interval, CI: 1.180–5.228, *P* = 0.017) and this result was no longer visible for OS.

**Table 2:** Multivariate Cox proportional hazard model of breast cancer-specific survival (BCSS) and overall survival (OS)

Variables		BCSS		OS	
		HRs (95% CI)	*P*^c^	HRs (95% CI)	*P*^c^
**Age (years)**	**18–49**	0.825 (0.701–0.971)	**0.020**	0.545 (0.480–0.618)	**< 0.001**
	**50–79**	Reference		Reference	
**Race**	**White**	Reference		Reference	
	**Black**	1.144 (0.946–1.383)	0.165	1.078 (0.944–1.232)	0.267
	**Other**^a^	0.610 (0.442–0.842)	**0.003**	0.668 (0.544–0.819)	**< 0.001**
**Marital status**	**Married**	Reference		Reference	
	**Not married^b^**	1.290 (1.111–1.497)	**0.001**	1.562 (1.417–1.722)	**< 0.001**
**Grade**	**I**	0.518 (0.329–0.816)	**0.005**	0.986 (0.834–1.166)	0.872
	**II**	Reference		Reference	
	**III and IV**	2.719 (1.779–2.670)	**< 0.001**	1.360 (1.941–2.403)	**< 0.001**
**Histology type**	**MBC**	1.304 (0.490–2.183)	0.931	1.518 (1.206–1.533)	0.188
	**IDC**	Reference		Reference	
**AJCC stage**	**I**	Reference		Reference	
	**II**	1.385 (0.976–1.965)	0.068	0.909 (0.730–1.131)	0.390
	**III**	1.530 (0.890–2.630)	0.124	0.923 (0.618–1.377)	0.694
**Tumor size (cm)**	**≤ 2**	Reference		Reference	
	**> 2 and ≤ 5**	1.917 (1.458–2.520)	**< 0.001**	1.776 (1.469–2.147)	**< 0.001**
	**> 5**	4.006 (2.862–5.607)	**< 0.001**	3.307 (2.580–4.239)	**< 0.001**
**Nodal status**	**0**	Reference		Reference	
	**1 to 3**	1.860 (1.499–2.308)	**< 0.001**	1.453 (1.260–1.676)	**< 0.001**
	**4 to 10**	3.011 (1.939–4.674)	**< 0.001**	2.405 (1.693–3.415)	**< 0.001**
	**> 10**	5.248 (3.440–8.005)	**< 0.001**	3.673 (2.607–5.175)	**< 0.001**
**Breast subtype**	**HR+/Her2−**	Reference		Reference	
	**HR+/Her2+**	0.582 (0.422–0.802)	**0.001**	0.827 (0.690–0.991)	**0.039**
	**HR−/Her2+**	1.449 (1.098–1.913)	**0.009**	1.247 (1.020–1.526)	**0.032**
	**Triple negative**	3.044 (2.553–3.628)	**< 0.001**	2.324 (2.055–2.628)	**< 0.001**
**Type of surgery**	**BCS**	Reference		Reference	
	**Mastectomy**	1.037 (0.875–1.229)	0.672	0.589 (0.769–0.959)	**0.007**
**Radiation**	**No**	1.796 (1.533–2.105)	**< 0.001**	2.396 (2.147–2.675)	**< 0.001**
	**Yes**	Reference		Reference	

Abbreviation: AJCC, American Joint Committee on Cancer; MBC, medullary breast carcinoma; IDC, invasive ductal carcinoma; Her2, human epidermal growth factor receptor 2; HR, hormone receptor; BCS, breast conserving surgery; HRs, hazard ratios; CI, confidence interval; BCSS, breast cancer-specific survival; OS, overall survival.

a Other includes American Indian/Alaskan native, and Asian/Pacific Islander.

b Not married includes divorced, separated, single (never married), unmarried or domestic partner and widowed.

c *P* value was adjusted by multivariate Cox proportional hazard regression model including all factors, as categorized in Table 2, and a bold type indicates significance.

### Survival estimates in matched group

**Figure 2:**
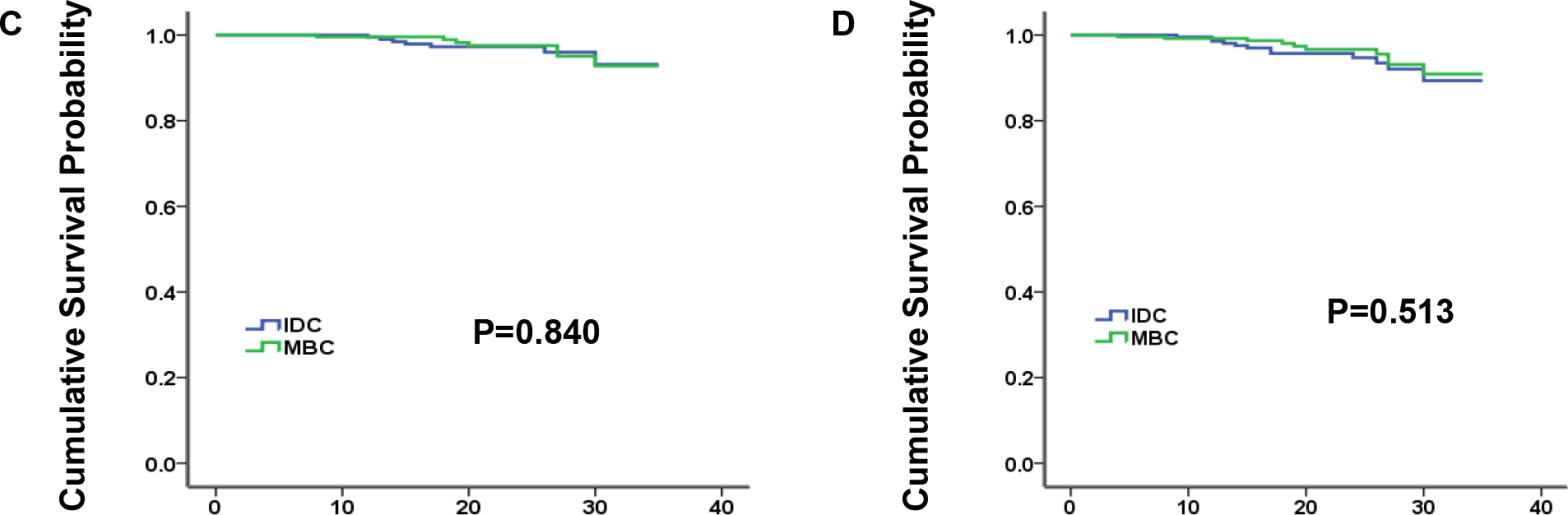
Kaplan-Meier plot and log-rank test compared breast cancer-specific survival (BCSS, C) and overall survival (OS, D) by histology for 1:1 matched group, medullary breast carcinoma (MBC) vs. invasive ductal carcinoma (IDC)

Considering the above confounding factors affecting breast cancer outcomes between MBC and IDC patients, we conducted a 1:1 (MBC/IDC) matched case–control analysis utilizing the propensity score matching method (Table 3). We obtained a group of 618 patients, including 309 patients for each histology type. For matched groups, no significant difference in characteristics was observed between MBC and IDC. Furthermore, we found that IDC histology no longer presented better prognosis for either BCSS or OS (Figure 2, *P* = 0.840 and *P* = 0.513 for BCSS and OS, respectively).

**Table 3:** Characteristics of patients with medullary breast carcinoma and invasive ductal carcinoma in 1:1 matched group

Characteristics		MBC (*n* = 309)		IDC (*n* = 309)		Total (*n* = 618)		*P*^c^
		No.	%	No.	%	No.	%	
**Median follow-up (months) (IQR)**		17 (9–27)		16 (8–25)		17 (8–26)		
**Age (years)**	**18–49**	130	42.1	106	34.3	236	38.2	**0.047**
	**50–79**	179	57.9	203	65.7	382	61.8	
**Race**	**White**	213	68.9	223	72.2	436	70.6	0.654
	**Black**	72	23.3	63	20.4	135	21.8	
	**Other**^a^	24	7.8	23	7.4	47	7.6	
**Marital status**	**Married**	185	59.9	194	62.8	379	61.3	0.457
	**Not married**^b^	124	40.1	115	37.2	239	38.2	
**Laterality**	**Left**	150	48.5	157	50.8	307	49.7	0.573
	**Right**	159	51.5	152	49.2	311	50.3	
**Grade**	**I**	2	0.6	2	0.6	4	0.6	1.000
	**II**	18	5.8	18	5.8	36	5.8	
	**III and IV**	289	93.5	289	93.5	578	93.5	
**AJCC stage**	**I**	126	40.8	126	40.8	252	40.8	1.000
	**II**	163	52.8	163	52.8	362	52.8	
	**III**	20	6.5	20	6.5	40	6.5	
**Tumor size (cm)**	**≤ 2**	141	45.6	148	47.9	289	46.8	0.666
	> **2 and ≤ 5**	155	50.2	145	46.9	300	48.5	
	**> 5**	13	4.2	16	5.2	29	4.7	
**Nodal status**	**0**	240	77.7	222	71.8	462	74.8	0.216
	**1 to 3**	54	17.5	75	24.3	129	20.9	
	**4 to 10**	10	3.2	8	2.6	18	2.9	
	**> 10**	5	1.6	4	1.3	9	1.5	
**Breast subtype**	**HR+/Her2−**	99	32.0	99	32.0	198	32.0	1.000
	**HR+/Her2+**	10	3.2	10	3.2	20	3.2	
	**HR−/Her2+**	27	8.7	27	8.7	54	8.7	
	**Triple negative**	173	56.0	173	56.0	346	56.0	
**Type of surgery**	**BCS**	174	56.3	158	51.1	332	53.7	0.197
	**Mastectomy**	135	43.7	151	48.9	286	46.3	
**Radiation**	**No**	153	49.5	165	53.4	318	51.5	0.334
	**Yes**	156	50.5	144	46.6	300	48.5	

Abbreviation: MBC, medullary breast carcinoma; IDC, invasive ductal carcinoma; AJCC, American Joint Committee on Cancer; Her2, human epidermal growth factor receptor 2; HR, hormone receptor; BCS, breast conserving surgery; IQR, interquartile range.

a Other includes American Indian/Alaskan native, and Asian/Pacific Islander.

b Not married includes divorced, separated, single (never married), unmarried or domestic partner and widowed.

c *P* value was calculated among all groups by the Chi-square test after matching on age, grade, AJCC stage, breast subtype, and a bold type indicates significance.

### Baseline characteristics and survival outcomes in triple-negative subgroup

**Figure 3:**
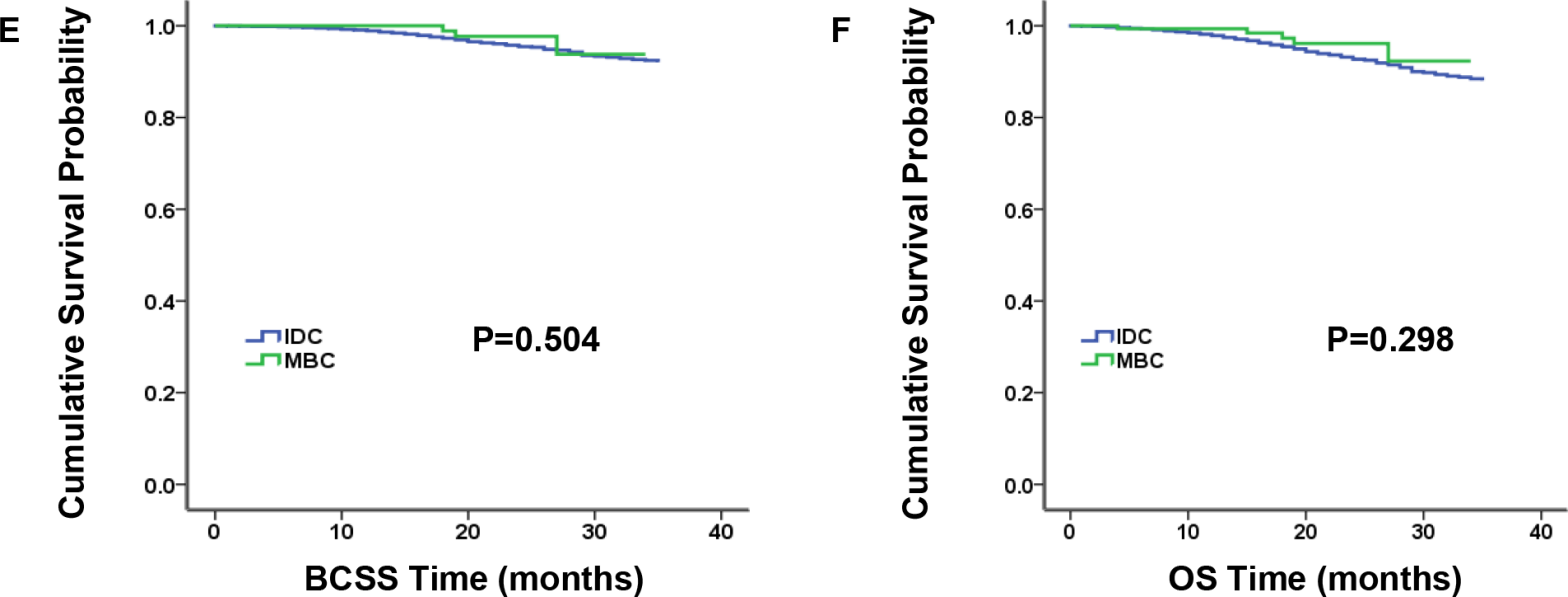
Kaplan-Meier plot and log-rank test compared breast cancer-specific survival (BCSS, E) and overall survival (OS, F) by histology for triple-negative breast cancer (TNBC) patients, medullary breast carcinoma (MBC) vs. invasive ductal carcinoma (IDC)

**Figure 4:**
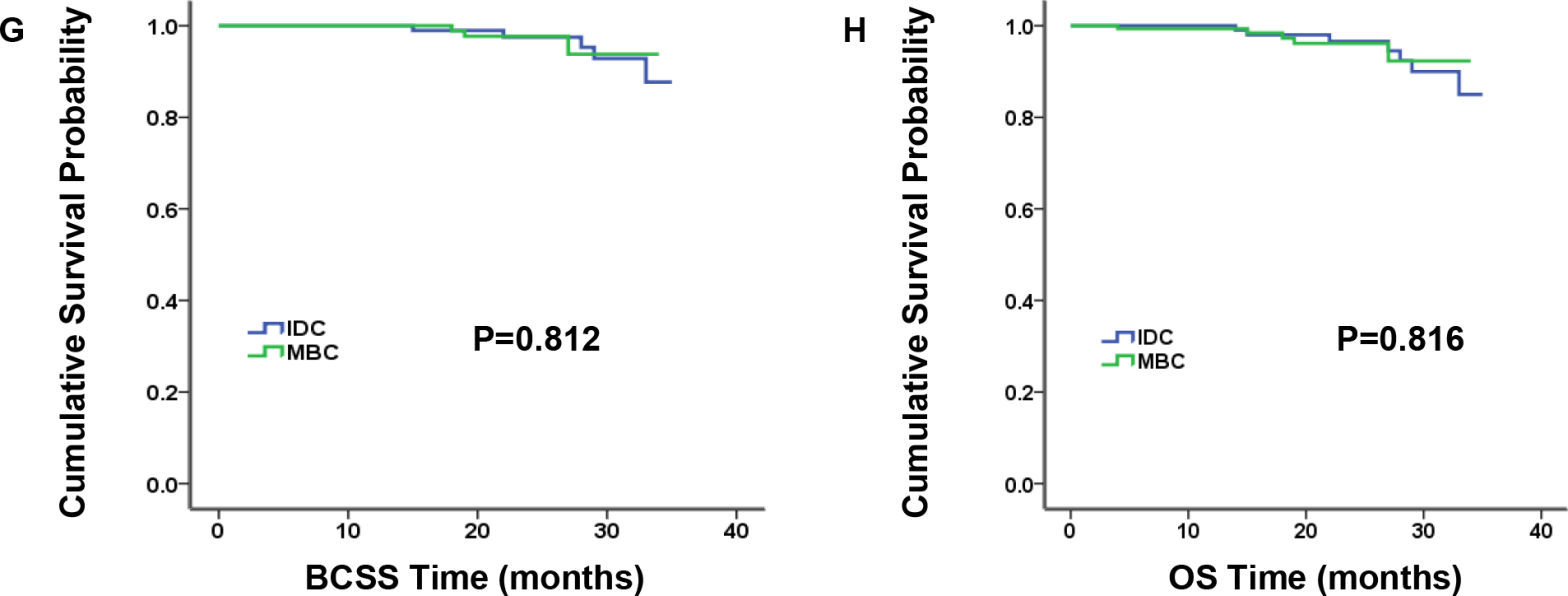
Kaplan-Meier plot and log-rank test compared breast cancer-specific survival (BCSS, G) and overall survival (OS, H) by histology for 1:1 matched triple-negative breast cancer (TNBC) patients, medullary breast carcinoma (MBC) vs. invasive ductal carcinoma (IDC)

MBC patients predominantly had TNBC according to molecular subtype (*P* < 0.001). Therefore, we analyzed characteristics and survival outcomes of the patients limited to TNBC subgroup, which contained 173 MBC patients and 11,056 IDC patients (Supplementary Table S2). And we came to some results in accordance with the entire population. For example, compared to TNBC-IDC patients, TNBC-MBC patients had younger age at diagnosis, larger proportion of black race and higher grade. Discriminately, nodal status and type of surgery become the significantly independent prognosis factors. However, AJCC stage and tumor size were no longer statistically different. In addition, the comparison of BCSS and OS by Kaplan-Meier curves presented similar survival for TNBC-MBC patients and TNBC-IDC patients (Figure 3, *P* = 0.504 and *P* = 0.298 for BCSS and OS, respectively). Furthermore, we matched 173 TNBC-MBC patients to 173 TNBC-IDC patients adjusted by age, AJCC stage and grade, utilizing the propensity score matching method. And we found BCSS and OS did not differ in the two groups (Figure 4, *P* = 0.812 and *P* = 0.816 for BCSS and OS, respectively).

### Stratification analysis with molecular subtype

In order to further investigate the effects of molecular subtype on breast cancer outcomes between MBC and IDC patients, we performed multivariate analysis, stratifying according to molecular subtype. As shown in Table 4, when hazard ratios (HRs) of the two histological groups were conducted, no differences in BCSS and OS were observed, suggesting that molecular subtype may be a principal confounder for MBC prognosis.

**Table 4:** Comparison of breast cancer-specific survival (BCSS) and overall survival (OS) between medullary breast carcinoma and invasive ductal carcinoma after subgroup analyses by multivariate Cox proportional hazard model

Subtype	BCSS			OS		
	Events No.	HRs (95% CI)	*P*^a^	Events No.	HRs (95% CI)	*P*^a^
**HR+/Her2−**			0.492			0.686
**MBC (*n* = 99)**	2	1.632 (0.404–6.595)		3	1.265 (0.406–3.945)	
**IDC (*n* = 60267)**	282	Reference		886	Reference	
HR+/Her2+			0.997			0.962
**MBC (*n* = 10)**	0	–		0	–	
**IDC (*n* = 9104)**	44	Reference		142	Reference	
**HR−/Her2+**			0.581			0.971
**MBC (*n* = 27)**	1	1.755 (0.239–12.892)		1	1.037 (0.144–7.494)	
**IDC (*n* = 4028)**	64	Reference		115	Reference	
Triple negative			0.742			0.535
**MBC (*n* = 173)**	4	0.847 (0.315–2.275)		6	0.775 (0.346–1.735)	
**IDC (*n* = 11094)**	329	Reference		541	Reference	

Abbreviation: MBC, medullary breast carcinoma; IDC, invasive ductal carcinoma; HR, hormone receptor; Her2, human epidermal growth factor receptor 2; HRs, hazard ratios; CI, confidence interval; BCSS, breast cancer-specific survival; OS, overall survival.

a *P* value was adjusted by multivariate Cox proportional hazard regression model including age, race, marital statuses, grade, tumor size, lymph node status, type of surgery, radiation and a bold type indicates significance.

## DISCUSSION

In this large cohort of patients, we sought to reveal the difference in characteristics and outcomes between MBC and IDC, and summarize the prognostic factors of MBC by utilizing SEER population-based data. Our findings indicated that MBC had unique clinicalpathological characteristics and it was inclined to demonstrate more aggressive behavior over IDC. However, MBC histology presented similar prognosis in both BCSS and OS compared to IDC after adjusting and matching the confounding factors. Further stratification analysis indicated that breast cancer molecular subtype may be a principal confounder for MBC prognosis. In addition, it was exposed that married status, grade, tumor size, lymph node status, breast subtype and radiation therapy were significantly associated with BCSS and OS.

In our study, compared with the IDC group, the MBC group presented a younger age at diagnosis, higher grade, more advanced stage, larger tumor size, and higher proportion of TNBC. These observations were partially in concordance with previous studies. For example, Anderson et al. conducted an age-specific incidence rate curve and showed that MBC rates increased rapidly until age 50 and then failed to increase, while IDC rates increased rapidly until age 50 years and rose more slowly thereafter [14]. Park et al. demonstrated that women with MBC presented rare LN metastasis, ER and PR negativity, advanced tumor grade, and nuclear pleomorphism [12]. Vo et al. reported that a MBC group had larger tumors than an IDC group [10]. However, patients with MBC in China demonstrated less aggressive tumor features such as lower tumor stage, smaller tumor size and a lower proportion of nodal involvement than IDC [5].

Previous studies have revealed that MBC has a more favorable prognosis than IDC. For example, Huober et al. found that 14-year distant recurrence-free interval (DRFI), and overall survival (OS) percents for medullary tumors and invasive ductal tumors of the full cohort were 76%, 64% and 66%, 57%, respectively [15]. However, some interesting findings observed in our study were that compared with IDC, MBC showed nearly the same outcomes as IDC in BCSS and OS after adjusting or matching the potential confounders. Collectively, these results implied that the MBC histological type was not an independent prognostic factor. Furthermore, among TNBC patients, MBC patients presented similar survival compared with IDC patients. Therefore, we speculated that breast cancer molecular subtype may be a principal confounder for MBC prognosis and it was further verified by stratification analysis with molecular subtype. In addition, we found that worse BCSS and OS for MBC compared to IDC were seen relevance to not married status, grade III and IV, a tumor size of > 5 cm, the increased number of lymph nodes, the subtype of TNBC and no receipt of radiation therapy.

Inevitably, our study had several limitations. In terms of follow-up data, it is the well-known fact that information regarding HER-2 expression in SEER database was not available until 2010. We were therefore compelled to focus on the short-term survival status after initial diagnosis and identify any prognostic factors and an inadequate follow-up time may give rise to the skewed results. And beyond that, differences in treatments received could influence differences in survival, but information regarding adjuvant or neoadjuvant chemotherapy is absent from the SEER database.

In conclusion, our investigations revealed that MBCs have unique clinicopathological characteristics, including a younger age at diagnosis, higher grade, more advanced stage, larger tumor size, and higher proportion of TNBC. However, MBC presented similar prognosis in both BCSS and OS compared with IDC. These results not only confer deeper insight into MBC but contribute to clinical practice that doctors ought to provide patients with MBC the same intensive treatment as those with IDC and, thereby may improve clinical management and outcomes.

## MATERIALS AND METHODS

### Patients

We used SEER *Stat version 8.2.1 to generate a case listing. We identified 84,764 eligible patients according to the following inclusion criteria: female, year of diagnosis from 2010 to 2012, age of diagnosis between 18 and 79 years, breast cancer as the first and only malignant cancer diagnosis, pathologically confirmed medullary breast carcinoma, not otherwise specified (MBC-NOS, ICD-O-3 8510/3) or invasive ductal carcinoma, not otherwise specified (IDC-NOS, ICD-O-3 8500/3), unilateral cancer, breast subtype, histological grades I to IV, AJCC TNM stages I-III, surgical treatment with either mastectomy or breast-conserving surgery, known ER, PR and HER2 statuses. We excluded patients who were diagnosed with breast cancer at death or by autopsy only and those with other first primary cancers, *in situ* disease, and no record of surgery type or radiation therapy. Patients diagnosed with breast cancer before 2010 were not included because the SEER database did not record data on HER2 status until 2010. Additionally, patients diagnosed with breast cancer after 2012 were not included because the database was only updated through December 31, 2012, and we wanted to ensure an adequate follow-up duration. We calculated follow-up times from January 1, 2010 to December 31, 2012.

### Statistical analysis

The demographics and clinical characteristics of incorporated cases were compared between MBC and IDC groups with the Chi square test. Kaplan-Meier method was performed to generate the survival curves, and the log-rank test was performed to compare the unadjusted BCSS and OS rates of patients with different histological subtype. BCSS was measured from the date of diagnosis to the date of breast cancer death. OS was defined as the time from the date of diagnosis to the date of death due to all causes (including breast cancer) or the last follow-up. Adjusted HRs with 95% CIs were calculated using Cox proportional hazard regression models in order to estimate the prognostic factors. These above statistical analyses were performed utilizing SPSS version 20.0 software package (IBM SPSS Statistics, Chicago, IL, US). In addition, we matched each MBC patient to 1 IDC patient on the following predetermined factors: age, AJCC stage, grade, breast subtype, utilizing psmatch2 in Stata, version 12.0 (StataCorp, College Station, TX) designed for the propensity score matching methods and tested the matching quality for the balance after the match. Two-sided *P*-value < 0.05 was considered statistically significant.

## SUPPLEMENTARY MATERIALS TABLES


