# Supplementary material for: Difference in characteristics and outcomes between medullary breast carcinoma and invasive ductal carcinoma: a population based study from SEER 18 database
Source: Oncotarget. 2016 Mar 17;7(16):22665–73. doi: 10.18632/oncotarget.8142 (PMC5008390; doi:10.18632/oncotarget.8142)
Supplement: Supplementary file 1 [file oncotarget-07-22665-s001.pdf]

Difference in characteristics and outcomes between medullary breast carcinoma and invasive ductal carcinoma: a population based study from SEER 18 database

Supplementary Materials

Supplementary Table S1: Univariate Cox proportional hazard model of breast cancer-specific survival (BCSS) and overall survival (OS)

Variables		BCSS		OS	
		HRs (95% CI)	*P*	HRs (95% CI)	*P*
**Age (years)**	**18–49**	1.303 (1.111–1.529)	**0.001**	0.690 (0.610–0.781)	**< 0.001**
	**50–79**	Reference		Reference	
**Race**	**White**	Reference		Reference	
	**Black**	2.033 (1.692–2.433)	**< 0.001**	1.623 (1.427–1.847)	**< 0.001**
	**Other[a]**	0.629 (0.456–0.867)	**0.005**	0.646 (0.528–0.792)	**< 0.001**
**Marital status**	**Married**	Reference		Reference	
	**Not married[b]**	1.542 (1.333–1.783)	**< 0.001**	1.788 (1.626–1.967)	**< 0.001**
**Laterality**	**Left**	Reference		Reference	
	**Right**	0.981 (0.848–1.134)	0.793	0.955 (0.868–1.051)	0.346
**Grade**	**I**	0.303 (0.194–0.476)	**< 0.001**	0.746 (0.634–0.879)	**< 0.001**
	**II**	Reference		Reference	
	**III and IV**	4.571 (3.798–5.500)	**< 0.001**	2.160 (1.941–2.403)	**< 0.001**
**Histology type**	**MBC**	2.483 (1.180–5.228)	**0.017**	1.518 (0.815–2.827)	0.188
	**IDC**	Reference		Reference	
**AJCC stage**	**I**	Reference		Reference	
	**II**	4.958 (4.006–6.138)	**< 0.001**	2.089 (1.872–2.332)	**< 0.001**
	**III**	15.963 (12.831–19.861)	**< 0.001**	4.568 (4.023–5.186)	**< 0.001**
**Tumor size (cm)**	**≤ 2**	Reference		Reference	
	**> 2 and ≤ 5**	4.930 (4.128–5.888)	**< 0.001**	2.389 (2.156–2.646)	**< 0.001**
	**> 5**	14.525 (11.740–17.970)	**< 0.001**	5.049 (4.348–5.862)	**< 0.001**
**Nodal status**	**0**	Reference		Reference	
	**1 to 3**	2.856 (2.395–3.405)	**< 0.001**	1.600 (1.429–1.792)	**< 0.001**
	**4 to 10**	5.952 (4.762–7.440)	**< 0.001**	2.827 (2.407–3.327)	**< 0.001**
	**> 10**	13.665 (11.000–6.976)	**< 0.001**	5.447 (4.606–6.441)	**< 0.001**
**Breast subtype**	**HR+/ Her2–**	Reference		Reference	
	**HR+/ Her2+**	1.003 (0.730–1.377)	0.987	1.037 (0.868–1.238)	0.690
	**HR–/ Her2+**	3.298 (2.519–4.318)	**< 0.001**	1.886 (1.555–2.289)	**< 0.001**
	**Triple negative**	6.131 (5.233–7.182)	**< 0.001**	3.221 (2.896–3.583)	**< 0.001**
**Type of surgery**	**BCS**	Reference		Reference	

	**Mastectomy**	2.496 (2.143–2.907)	**< 0.001**	1.765 (1.603–1.943)	**< 0.001**
**Radiation**	**No**	1.689 (1.458–1.957)	**< 0.001**	2.201 (1.993–2.429)	**< 0.001**
	**Yes**	Reference		Reference	

Abbreviation: AJCC, American Joint Committee on Cancer; MBC, medullary breast carcinoma; IDC, invasive ductal carcinoma; Her2, human epidermal growth factor receptor 2; HR, hormone receptor; BCS, breast conserving surgery; HRs, hazard ratios; CI, confidence interval; BCSS, breast cancer specific survival; OS, overall survival.
[a]Other includes American Indian/Alaskan native, and Asian/Pacific Islander.
[b]Not married includes divorced, separated, single (never married), unmarried or domestic partner and widowed.

Supplementary Table S2: Characteristics of patients with triple negative tumors, MBC vs. IDC

**Characteristics**		**MBC (*n* = 173)**		**IDC (*n* = 11056)**		**Total (*n* = 11229)**		***P*** [c]
		**No.**	**%**	**No.**	**%**	**No.**	**%**	
**Age (years)**	**18–49**	75	43.4	3313	30.0	3388	30.2	**< 0.001**
	**50–79**	98	56.6	7743	70.0	7841	69.8	
**Race**	**White**	114	65.9	8103	73.3	8217	73.2	**0.007**
	**Black**	50	28.9	2153	19.5	2203	19.6	
	**Other**[a]	9	5.2	800	7.2	809	7.2	
**Marital status**	**Married**	100	57.8	6705	60.6	6805	60.6	0.448
	**Not married**[b]	73	42.2	4351	39.4	4424	39.4	
**Laterality**	**Left**	90	52.0	5721	51.7	5811	51.7	0.942
	**Right**	83	48.0	5335	48.3	5418	48.3	
**Grade**	**I**	2	1.2	185	1.7	187	1.7	**< 0.001**
	**II**<br>**III and IV**	6<br>165	3.5<br>95.4	1817<br>9054	16.4<br>81.9	1823<br>9219	16.2<br>82.1	
**AJCC stage**	**I**	72	41.6	4768	43.1	4840	43.1	0.136
	**II**	89	51.4	5059	45.8	5148	45.8	
	**III**	12	6.9	1229	11.1	1241	11.1	
**Tumor size (cm)**	**≤ 2**	80	46.2	5587	50.5	5667	50.5	0.073
	**> 2 and ≤ 5**	86	49.7	4672	43.2	4758	42.4	
	**> 5**	7	4.0	797	7.2	804	7.2	
**Nodal status**	**0**	142	82.1	7743	70.3	7915	70.5	**0.007**
	**1 to 3**	22	12.7	2342	21.2	2364	21.1	
	**4 to 10**	7	4.0	568	5.1	575	5.1	
	**> 10**	2	1.2	373	3.4	375	3.3	
**Type of surgery**	**BCS**	103	59.5	5633	50.9	5736	51.1	**0.025**
	**Mastectomy**	70	40.5	5423	49.1	5493	48.9	
**Radiation**	**No**	82	47.4	5497	49.7	5579	49.7	0.545
	**Yes**	91	52.6	5559	50.3	5650	50.3	

Abbreviation: MBC, medullary breast carcinoma; IDC, invasive ductal carcinoma; AJCC, American Joint Committee on Cancer; BCS, breast conserving surgery.
[a]Other includes American Indian/Alaskan native, and Asian/Pacific Islander.
[b]Not married includes divorced, separated, single (never married), unmarried or domestic partner and widowed.
[c]*P* value was calculated among all groups by the Chi-square test, and a bold type indicates significance.
